# Recent Developments in the Diagnosis and Treatment of Disseminated and Rare Melanoma with Radiotheranostics

**DOI:** 10.1021/acsptsci.5c00736

**Published:** 2026-06-05

**Authors:** Rüdiger M. Exner, Jason S. Lewis, Lisa Bodei, Naga Vara Kishore Pillarsetty

**Affiliations:** † Department of Radiology, 5803 Memorial Sloan Kettering Cancer Center, New York, New York 10065, United States; ‡ Molecular Pharmacology Program, Memorial Sloan Kettering Cancer Center, New York, New York 10065, United States; § Department of Pharmacology, Weill Cornell Medicine, New York, New York 10065, United States; ∥ Department of Radiology, Weill Cornell Medicine, New York, New York 10065, United States

**Keywords:** radiotheranostics, radioligands, nuclear imaging, radiotherapy, melanoma, rare cancers

## Abstract

Disseminated melanoma, particularly of rare subtypes such as mucosal or uveal melanoma, is a difficult to treat, deadly disease characterized by poor median survival and resistance to a wide range of treatments. Despite new approvals, such as novel immunotherapeutics, there is a significant need for effective therapeutic modalities. Given the successes in the development of targeted radiopharmaceutical diagnostic agents and therapies for other cancers (the so-called theranostics), assessing the efficacy of these strategies in the context of melanoma appears as the next logical step. Here we aim to provide an overview of recent advances in the development of preclinical and clinical melanoma-specific radiopharmaceuticals. Where applicable, we will highlight their relevance in the context of rare subtypes of melanoma.

## Graphical abstract

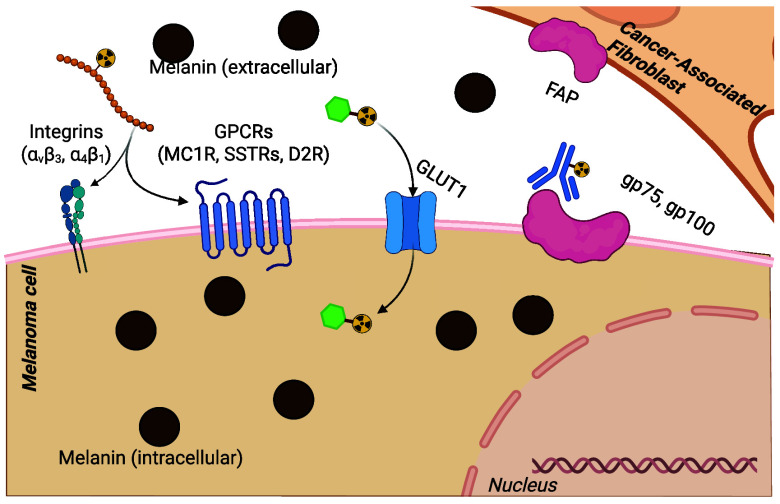

Early diagnosis is the single-most important prognostic factor in the survival rates of patients with melanoma. While relative survival across all stages for cutaneous melanoma is larger than 90%, this number is primarily influenced by early diagnosis—between 2012 and 2021 almost 80% of patients were diagnosed with local disease.^1,2^ This facilitates full surgical resection of cancerous tissues. Survival rates for patients diagnosed at later stages drop significantly—patients with regional metastases face 5-year survival rates of 74%, while patients with distant metastases at diagnosis face 5-year survival rates of ca. 35%.^3,4^ This overall picture becomes more complex once other, rare subtypes of melanoma are considered. Acral lentiginous melanoma (AM), i.e., melanoma that develops on the palms of the hands, soles of the feet or underneath finger- or toenails accounts for ca. 2% of all melanoma cases diagnosed each year.^5^ It has a higher tendency to go un- or misdiagnosed until later stages and may be more aggressive than cutaneous melanoma (CM), highlighted by lower 5-year survival rates even in early stages.^5,6^ Patients with mucosal melanoma (MM), i.e., melanoma that develops in mucous membranes, most commonly in the sinonasal space, are typically diagnosed at later stages as the primary tumor is often not readily visible.^7^ It accounts for 0.8–3.7% of all melanoma diagnoses in caucasians.^7,8^ For uveal melanoma (UM), early stages of the disease may be completely asymptomatic, or present with unspecific, easily overlooked or misdiagnosed symptoms.^9,10^ UM constitutes approximately 4–5% of all melanoma, with approximately half of all diagnosed patients eventually developing distant metastasis.^10^ The primary organ for metastatic spread of UM is the liver with 90% of patients developing hepatic metastases.^9,10^ In addition, metastasis to the lungs and bones is frequently observed.^9,10^ Noticeably, even with early diagnosis, approximately 55% of all UM patients will go on to develop metastases within 15 years from the initial diagnosis.^9,10^ This is believed to occur due to early seeding through the bloodstream, followed by the development of dormant micrometastases.^11^ Once metastases are diagnosed, UM patients face poor survival rates and low median survival of ca. 21 months.^9,12^ Despite approvals of highly specific chemotherapeutics, such as BRAF V600E inhibitors (vemurafenib, encorafenib and dabrafenib) and immune checkpoint inhibitors (either as single or combination treatments), which have revolutionized the treatment regimen for many cancer patients prognoses remain grim, with the median survival for patients with metastatic melanoma merely increasing to 21.1 months by 2018.^13^ In many cases, patients with melanoma will eventually develop resistance to these types of treatments.^14–16^ For instance, while ca. 50% of melanoma patients possess the BRAF V600E mutation, the majority of patients treated with vemurafenib will eventually show signs of disease progression due to innate or acquired resistance.^17^ In these patients, melanoma cells or the tumor microenvironment adapt to create alternative survival mechanisms. These include the secretion of hepatocyte growth factor (HGF) by stromal cells, overexpression of the platelet-derived growth factor receptor β (PDGFRB) and a mutation in a second oncogene, NRAS, which reactivates the original BRAF survival mechanism.^18^ More recent approaches, such as the use of immune checkpoint inhibitors (ICIs) or combination therapies have represented a breakthrough in the treatment of melanoma.^19^ However, despite increasimmune checkpoint inhibitors (ICIs) or combination therapieses in overall survival and progression-free survival, the objective response-rates (ORRs) are limited, particularly for single-agent treatment and rare melanoma subtypes.^16,19–22^ In a study in 676 patients, using the CTLA4-inhibitor ipilimumab either as a single-agent or in combination with a gp100 (glycoprotein 100) peptide vaccine, a disease control rate of 28.5% was observed.^23^ The median overall survival for patients treated with ipilimumab alone in this study was a mere 10.1%. Similar results have been reported for patients with AM, in which the response rates were determined to be 11.4–25%.^24^ Anti-PD1 and Anti-PD-L1 antibodies or combination approaches (PD-1/CTLA-4) generally show slightly better response rates in melanoma.^19–21^ However, many patients eventually develop resistance to the therapy with ICIs, leading to progression of disease despite ongoing treatment.^16^ In patients with UM, in which even dual-ICI treatment frequently fails (objective response rates of ca. 12.4%)^22^ the approval of tebentafusp, a first-in-class fusion protein comprising of an ScFv targeting a processed peptide of glycoprotein 100 (gp100, also known as the premelanosome protein, PMEL) presented by the human leukocyte antigen (HLA) and a T-cell engager has shown promising results, increasing median overall survival of patients with disseminated disease from approximately 17 months to 21 months.^9,12^ In light of these challenges, it is evident that there is a necessity for a new generation of diagnostic and therapeutic modalities for patients with disseminated melanoma. While external beam radiation and plaque brachytherapy have been investigated in-depth and are used in the treatment of localized melanoma, including rare subtypes,^25–27^ targeted radiopharmaceutical therapy has mostly been limited to a few select antigens. Most of this work has so far remained in preclinical stages, with only a few imaging agents and three therapeutic agents, [^131^I]­ICF01012, [^212^Pb]­MTI201 and [^212^Pb]­VMT01 being assessed in a clinical setting.^28–32^ These considerations led us to review and highlight current advances in this field and to highlight potential future directions for the development of theranostics targeting the various melanoma subtypes.

## Radiosensitivity of Melanoma Cells

To this day, melanoma holds the reputation of being radioresistant. This was originally based on the rationale that the increased production of melanin offers some level of protection against ionizing radiation.^33^ However, while the increased melanin-production of melanoma cells by itself offers some protection against ionizing radiation, several authors have pointed out that a more nuanced view is necessary.^33–36^ Many commonly used melanoma cell lines may indeed be considered radiosensitive. In the clinic, external beam radiation and stereotactic radiosurgery (SRS) are frequently used and effective treatment modalities.^25,27,33,35^ In patients with brain metastases SRS achieves high control rates,^37^ and combination approaches of SRS and ICIs have been assessed in the clinic.^38,39^ An in-depth understanding of the determinants of radiosensitivity or radioresistance in melanoma will be necessary moving forward, to improve the identification of patient subpopulations which are likely to benefit from radiation treatments. To date, many aspects remain elusive due to the complex interplay of various factors, such as tumor hypoxia, DNA damage signaling and repair proteins, the tumor microenvironment, redox balance and melanogenesis among others.^40^ When targeted radiopharmaceutical therapy is considered, additional factors play a role, such as the homogeneity of antigen-expression, the nature of the decay and the nature of metastasis (e.g., small micrometastases are thought to absorb less dose from treatment with highly energetic, long-range β-emitters, leading to the frequent observation of the difficult eradication of diffuse marrow infiltration in patients treated with Pluvicto).^41^ Nevertheless, based on the clinical successes with external beam approaches and recent developments in the field of targeted radiotherapies for other cancers, it appears prudent to consider the development of such therapies for the therapy of disseminated melanoma.

### Surface Antigens for the Nuclear Imaging and Radiopharmaceutical Therapy in Melanoma

While most work in the field of targeted radiotherapy in melanoma has focused on external beam approaches, such as SRS, efforts have been undertaken to develop targeted agents against a variety of antigens. While in most cases melanomas tend to be FDG-avid, false-positives with FDG, as well as a lack of FDG-avidity can affect results and lead to incorrect evaluation. For example, the high liver and brain uptake associated with [^18^F]­FDG may negatively affect the detection of metastases in these organs.^30,42–45^ Particularly in patients with ocular melanoma, liver metastases tend to have lower SUVmax than those of patients with CM, leading to inaccurate staging or evaluations.^46^ As such, there is a clear clinical need for more personalized approaches to allow for adequate staging and therapy monitoring. Given the lack of response to many types of treatments, the consideration of radiopharmaceutical therapy appears prudent in these cases. Here we aim to give an overview over the developmental, preclinical and clinical work that has been performed toward the use of melanoma-specific diagnostic and theranostic agent, and where possible appraise their utility for the monitoring or treatment of the various rare subtypes of melanoma.

### The Melanogenic Pathway as a Source of Target Antigens

The melanogenic pathway would be an ideal source of target antigens for the therapy of melanoma, as it is well conserved throughout disease progression and many of its proteins tend to be significantly upregulated, relative to healthy skin cells. However, a central challenge in selecting antigens from the melanogenic pathway is the fact that most members of this pathway typically do not show cell-surface expression or are only expressed transiently on cell surfaces. As such, most work in this field has been focused on the melanocortin-1-receptor (MC1R) which is the only melanogenesis-related protein canonically trafficked to the cell surface of melanocytes and frequently overexpressed in melanoma.^47,48^ Particularly mucosal and uveal melanoma are known to frequently (over)­express MC1R, rendering it an attractive target for theranostic applications.^47–49^ However, overexpression of proteins that are commonly thought of as ‘intracellular’, transient cycling through the membrane and aberrant processing or trafficking in cancer cells may cause increased cell-surface availability.^50,51^ Examples of this can be found in recent advances in targeting of glycoprotein 75 (gp75, also known as tyrosinase-related protein 1, TYRP1) and glycoprotein 100 (gp100, also known as the premelanosome protein, PMEL) or fragments thereof.^9,12,50,52–56^

### Melanocortin-1-Receptor

A significant body of work on exploiting MC1R as a target for radiotheranostics has been published, including both preclinical and clinical developments, as well as several in-depth review articles outlining advances in the field.^28,29,48,57–65^ Like the well-known somatostatin receptor, which is a classic example, this G-protein coupled receptor is a prominent target, due to its overexpression across all subtypes of melanoma,^47,49,66^ and the fact that it is canonically cell-surface bound.^47^ It can be targeted with various small peptides and as such lends itself particularly well to radiopharmaceutical therapy. These small peptides are generally derivatives of MC1R’s natural agonist, the α-melanocyte stimulating hormone (α-MSH).^67^ The main limitation of α-MSH is the lack of biological stability, with a short plasma half-life of approximately 20 min in patients (β-phase elimination half-life).^68^ To improve upon this, various modifications of the peptide sequence have been successfully integrated, most of which were inspired by afamelanotide (melanotan I) and melanotan II.^67^ These range from the introduction of noncanonical amino acids to cyclization,^48,58,60^ either by formation of disulfide bonds or metal complexes involving cysteine residues,^69,70^ or by formation of a lactam, via ring-closure with the ε-amine group of a lysine residue.^60^ Both linear as well as cyclized peptides have been assessed, with the identified minimal peptide sequence for efficient binding being His-Phe-Arg-Trp (HFRW).^67^ Typically, the phenylalanine in this sequence is exchanged for the D-stereoisomer (HfRW), as this inversion was found to not affect binding and offer increased stability of the resulting peptides. Noticeably, many synthetic MC1R agonists have proven to be less selective than endogenous α-MSH, with significant off-target binding to the other melanocortin receptors MC3R-MC5R being observed.^71^ These are endogenously expressed in a variety of tissues and are responsible for the side-effects observed in approved treatments with MC1R agonists (e.g., drugs against phototoxicity such as afamelanotide).^67,71^ New generations of MC1R inhibitors achieve remarkable selectivity, often exceeding that of natural α-MSH.^71^ However, the currently used radiopharmaceuticals in this space, which are based on melanotan II, already show promising biodistribution and dosimetry in both preclinical models and patients.^28,29,57,58,61–63,72–74^ As such, it is unclear whether higher selectivity is required in this context. Initial work investigating the use of MC1R agonists as theranostics for melanoma, focused on linear derivatives, often stabilized by introduction of a norleucine (Nle) residue, as well as N- and C-terminal modifications. DOTA-NAPamide features an N-terminal acetate residue, followed by Nle and a C-terminal amide, Ac-Nle-DHfRWGK­(DOTA)-NH_2_.^75–77^ As an alternative to NAPamide, MC1RL was developed, which instead of the N-terminal acetate features an N-terminal phenylbutanamide.^48^ While these gave promising results, both for imaging and therapy applications, the tumor-to-kidney ratios were relatively low, leading to an improved version featuring a more lipophilic linker, thus rerouting this radiopharmaceutical to hepatic clearance.^78^ Alternatively, the cyclized peptides, demonstrated significantly improved overall dosimetry and higher tumor uptake.^60,79^ Initial generations of these were cyclized through rhenium or disulfide-bridged derivatives which offered improved tumor-to-tissue ratios and better retention.^69,70^ Besides these, lactam-derivatives based on melanotan II, featuring DO3A-chelators, developed by the groups of Benard and Miao have proven to be excellent MC1R targeting moieties.^58–62,72,79^ Especially the work of Guo et al., who pioneered the lactam-based derivatives with reduced ring-size and investigated the influence of linker-modification,^80,81^ has been instrumental in further advancing the field and led to the DOTA-CycMSH_hex_-derivatives that are at the forefront of this field today, leading to the first in-human trials of [^68^Ga]­Ga-DOTA-GGNle-CycMSHhex.^72^ Using this same cyclized peptide backbone, the group of Uehara has recently developed an MC1R-inhibitor featuring their neopentyl glycol-based prosthetic group and were thus able to produce targeted radiotherapeutics incorporating Iodine-131 and Astatine-211 with high biochemical stability.^63^ Beyond this, extensive optimization into the functionality of the linker have been undertaken in an attempt to further improve tumor uptake, for instance by incorporating albumin binders, or designing linkers which can be cleaved by renal brush border enzymes.^61,82^ The structures of α-MSH, along with selected linear and cyclized peptides targeting MC1R are provided in Figure 1. From a clinical perspective, MC1R presents an excellent target, with expression commonly observed in primary tumors and metastases.^47^ In fact, expression levels in metastases are often found to be higher than those of primary tumors.^47^ In addition, MC1R is commonly found in all subtypes of melanoma, including cutaneous, mucosal and uveal melanoma (with over 94% of UM metastases expressing MC1R).^49^ The corresponding targeting agents show promising dosimetry and have been tested in several clinical studies for imaging and therapy purposes. Two Ga-68 labeled agents, [^68^Ga]­Ga-DOTA-GGNle-CycMSH_hex_ and [^68^Ga]­Ga-CCZ01048 have been assessed in comparison studies with [^18^F]­FDG in patients.^57,72^ While FDG-PET was noninferior to [^68^Ga]­Ga-DOTA-GGNle-CycMSH_hex_, the study highlighted the variable MC1R expression between lesions and demonstrated that brain metastases could be detected more readily with the MC1R-speicifc tracer, due to high brain uptake of [^18^F]­FDG. The subsequently developed [^68^Ga]­Ga-CCZ01048, demonstrated fast systemic clearance and higher SUV values, as compared to [^18^F]­FDG, thus outperforming it. Figure 2 shows PET images of a patient with disseminated MM of the perineal region highlighting local recurrence in the perineal and perirectal regions, as well as pulmonary metastases, which are readily delineated with the MC1R-targeting agent, although uptake here was likely insufficient for therapeutic applications. A representative of the lactam-cyclized peptides, labeled with the α-emitting Pb-212, [^212^Pb]­VMT01 was recently assessed in a phase I/IIa study.^28^ In this study, patients were randomized into low- and high-dose groups, receiving three intravenous injections of either 111 MBq (3 mCi) or 185 MBq (5 mCi), respectively. Interim results indicated that only patients in the ‘low’ dose group showed a response to therapy, while patients in the ‘high’ dose group did not.^28^ This was proposed to be related to the involvement of immune infiltration, following the radiotherapy, and will be further explored in a follow-up trial involving a low-dose treatment regimen complemented by administration of the PD-1 inhibitor nivolumab, which was previously demonstrated to potentiate therapeutic efficacy in preclinical models.^28,29^ In a small study including seven patients with uveal melanoma, [^225^Ac]-MTI-201 (DOTA-MC1RL) was assessed in patients with metastatic uveal melanoma.^74,83^ In the patient population of this initial study, stable disease was observed in two patients after a single administration of up to 76 μCi (2.812 MBq). The radiopharmaceutical was well tolerated at these dose levels, with no dose-limiting or grade 4 toxicity being observed.^83^ It may be of interest to consider the pharmacological upregulation of MC1R in the context of clinical therapy studies, to further improve uptake in tumors, as has been successfully demonstrated preclinically, using BRAF, MAPK and HDACs inhibitors.^73^ It should be noted here that the MC1R-binding peptides presented in this subchapter are agonists. In other target antigens, such as bombesin receptors, it was reported that antagonists give superior performance, when compared to agonistic radiopharmaceuticals.^84,85^ As such, it may be of interest to explore the performance of MC1R antagonists for imaging and therapy applications. Furthermore, in murine models, high specific activities were found to significantly improve uptake,^62^ such that many groups describe procedures in which even MC1R agonists labeled with radioisotopes of metals, such as Ga-68, are further purified by semipreparative HPLC to remove the unlabeled peptide-conjugates.^59,77^ This may be a result of low receptor densities, even in high expressing models such as B16–F10 cells (21,687 ± 4171sites/cell).^59^ In addition, a caveat of targeting MC1R may be, that while it is also frequently expressed in amelanotic melanoma, it typically appears in one of various mutated forms, such as the ones associated with phenotypes such as red hair. These mutations tend to lead to reduced ability to bind α-MSH,^86^ and are by extension, likely to be less suitable targets for the various MC1R-targeting radiopharmaceuticals as well and could potentially lead to reduced therapeutic efficacy through antigen escape.

**Figure 1:**
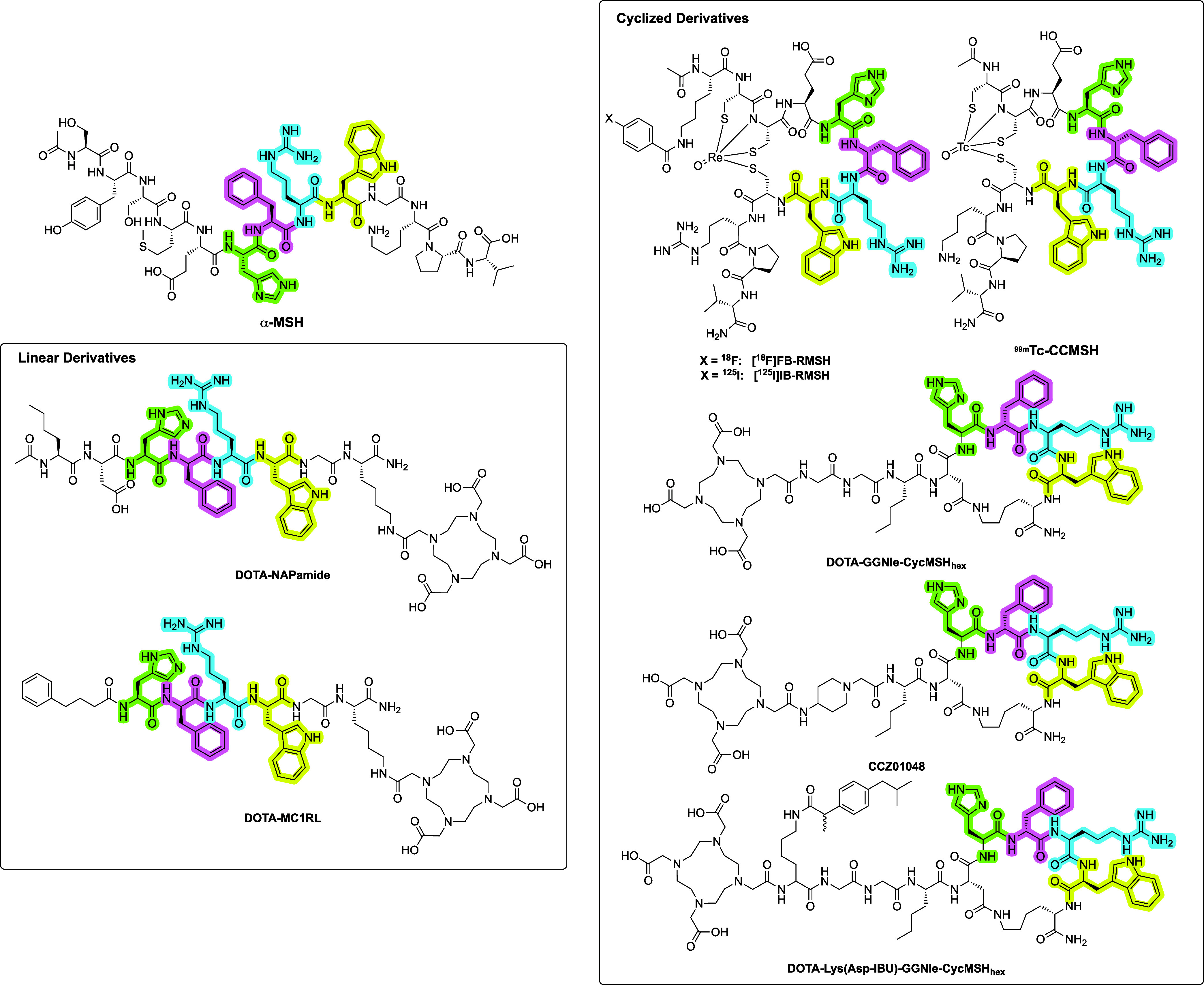
Structure of a-MSH (top left) and selected linear (left) and cyclized (right) MC1R binding peptides. Minimal binding sequence (HFRW/HfRW) highlighted.

**Figure 2:**
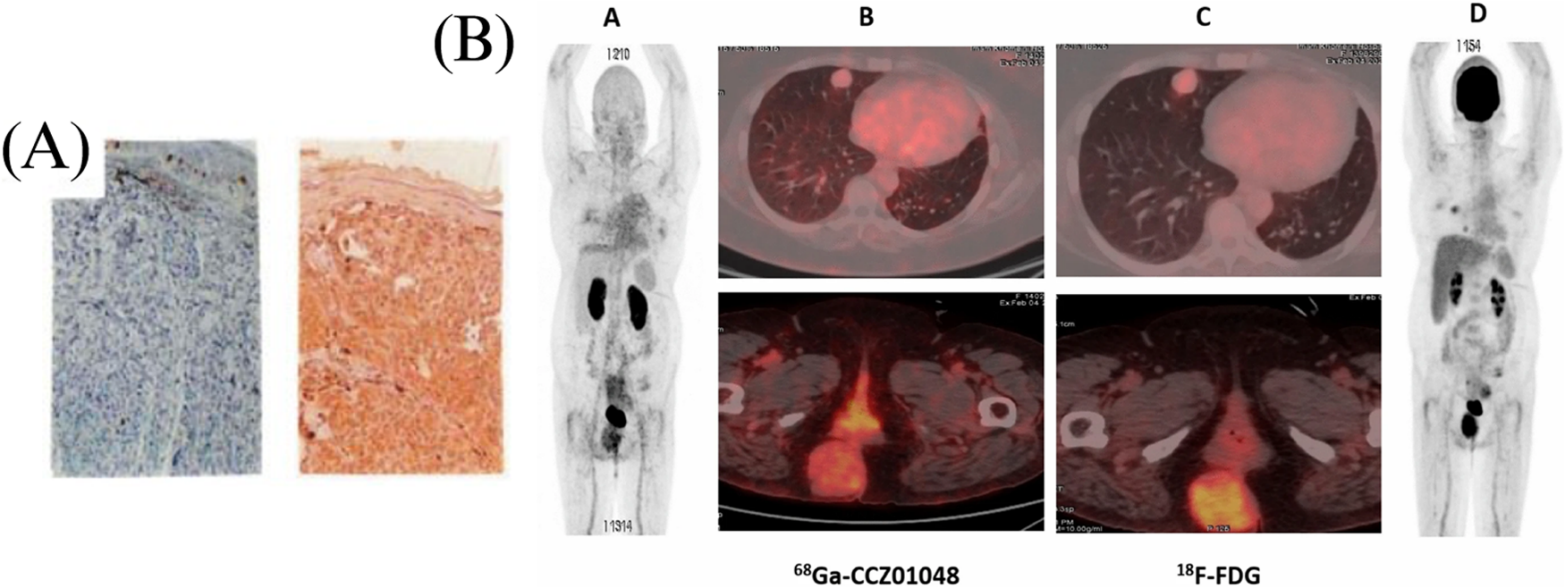
(A) immunohistochemical detection of MC1R in normal naevus (left) and primary melanoma (right) highlighting higher expression levels in metastatic tissues. Adapted from ref 66 (Salazar-Onfray et al., 2002), licensed under a CC BY-NC-SA 3.0 license. (B) PET images of [^68^Ga]­Ga-CCZ01048 (left) and [^18^F]­FDG (right), highlighting the favorable biodistribution and tumor uptake of MC1R-targeted theranostic agents. Reproduced with permission from ref 57 (Zolghadri et al., 2025), licensed under a CC BY-NC-ND 4.0 license.

### Glycoprotein 75—Tyrosinase-Related Protein 1

Tyrosinase-related protein 1 (TYRP1, also known as glycoprotein 75, gp75) is a well-established target for all subtypes of melanoma.^50,51,53,54,87^ In particular, mucosal and uveal melanoma are found to commonly overexpress this protein. TYRP1 is a zinc metalloenzyme with a role in melanosome maturation.^87–89^ Its exact role in humans is not well understood, as some studies suggested that it does not possess the DHICA oxidase activity observed for murine TYRP1.^88,90^ Nevertheless, TYRP1 has been shown to be a negative prognostic marker in human melanoma and appears to be well conserved throughout disease progression.^91^ Moreover, it is frequently overexpressed in rare subtypes, such as mucosal and uveal melanoma.^50,54^ While recently some attempts were made to use gp75 as a melanoma-specific target antigen in a variety of preclinical and clinical settings by leveraging CAR-T cells, bispecific T cell engagers or monoclonal antibodies,^50,53,54^ no radiopharmaceutical has entered the clinical space at the time of writing. However, preclinical work into the SPECT imaging of TYRP1 using the murine antibody TA99, as well as our own recent work using the human antibody flanvotumab for the PET/CT imaging and targeted radiotherapy of this target has recently revealed promising results in syngeneic and xenograft models of melanoma.^52,92^ Noticeably, therapy with the α-emitting radioisotope Ac-225 with TA99 appeared to give lackluster therapeutic response, while treatment with the β-emitting radioisotope Lu-177 reduced tumor growth and increased median survival significantly.^52,55^ An interesting aspect of the study featuring Ac-225 by Constanzo et al. is that in different backgrounds, differential therapeutic response was observed. While a syngeneic, fully immunocompetent model of B16–K1 cells in C57BL/6 mice showed some response to the radioimmunoconjugate treatment, changing the background to athymic nude mice abrogated the therapeutic response. Given the fact that TYRP1 is commonly expressed across all melanoma subtypes we believe that it is a target antigen which warrants further studies.

### Melanin

Melanin itself, or more specifically eumelanin, has been explored as a target for the PET-imaging of melanoma.^30,32,93^ Due to the hyperpigmentation of many melanoma lesions, and the secretion of melanin into the tumor microenvironment,^94^ selective binding of this biopolymer can achieve significant uptake and signal-to-background ratios as demonstrated by several groups over the last decades.^93,95,96^ As the field of melanin-specific imaging agents has recently been reviewed by Zhang et al.,^93^ we intend to only provide a brief overview here. Many of the melanin-binding SPECT and PET radiotracers feature an N-[2-(diethylamino)­ethyl]-motif in addition to an aromatic core. They can broadly be considered as being inspired by the antimalaria drug chloroquine and the antipsychotic drug chlorpromazine, radiolabeled derivatives of which were found to accumulate in human malignant melanoma.^97,98^ The mechanism by which these probes bind to melanin has not been investigated in detail, but it has been suggested that a combination of ionic interactions between the carboxylate groups of the eumelanin pigment and the tertiary amine functionalities, and potentially π-π interactions (in derivatives with aromatic motifs) play a central role.^99^ Figure 3 shows a selection of several melanin-binding radiotracers featuring C-11, F-18, Ga-68, Tc-99m and I-123/125/131.^32,100–108^ While initial work focused on the development of SPECT imaging and potential therapeutic agents with iodine radionuclides, more recent work shifted the attention toward PET-imaging, with the development of F-18 and Ga-68 based probes to allow for routine acquisition of high-resolution 3D-images. Some of this preclinical work has been translated to the clinic, with various trials, including ones specific to ocular melanoma starting in the 90s,^109^ however no approved imaging agent has entered the market at the time of writing. Among the ones that have been assessed in clinical trials, the PET tracers [^18^F]­MEL050, [^18^F]­PFPN, [^18^F]­DMPY2 and [^18^F]­P3BZA have been studied more recently.^30,31,44,45,95,110,111^ In a comparative study between [^18^F]­MEL050 and FDG, it was found that the heterogeneity in melanin production between various metastases led to poor performance, with only 48% of metastases detected by FDG-PET being delineated with [^18^F]­MEL050 (Figure 4A).^111^ In contrast, [^18^F]­PFPN performed considerably better in patients with various subtypes of melanoma, being able to detect lesions which were not detected when using FDG. Figure 4B shows the [^18^F]­FDG and [^18^F]­PFPN PET-scans of a patient who underwent removal of a choroidal melanoma 19 months prior to imaging, highlighting the potential of these melanin-binding probes in multiple subtypes of melanoma.^45^ [^18^F]­PFPN has moreover demonstrated promising preliminary results in patients with ocular and orbital melanoma, outperforming [^18^F]­FDG which was limited by lower SUV_max_ and higher uptake in periocular tissues, particularly the brain. Figure 4C shows PET/CT images of another fluorine-18 based, melanin-targeting probe: [^18^F]­DMPY2 in a patient with melanoma, in comparison to those obtained with [^18^F]­FDG, demonstrating higher SUVmax in the inguinal lymph node.^30^ Beyond the development for imaging applications, some groups have evaluated the potential of using melanin-binding radiopharmaceuticals for therapeutic purposes.^32,112,113^ These agents, to the best of our knowledge, all feature the β^–^-emitting isotope iodine-131. One of these agents, [^131^I]­ICF01012 is currently being assessed in clinical trials.^32^ While the preclinical and clinical results with small-molecule melanin-based tracers are overall promising and will make for more disease-specific markers than the current gold-standard FDG, they inevitably fail to detect amelanotic lesions.^111^ In addition, melanin in healthy organs and tissues, such as the skin, eyes and brain (basal ganglia) would also be targeted, which needs to be taken into account.^32^ In this context, it may be of interest to evaluate some of these probes toward their ability to bind to pheomelanin, which may still be found in amelanotic melanoma.^114^ The difference in performance between the various, structurally related compounds is remarkable and highlights the need for in-depth structure activity relationship studies, to identify the most promising candidates.

**Figure 3:**
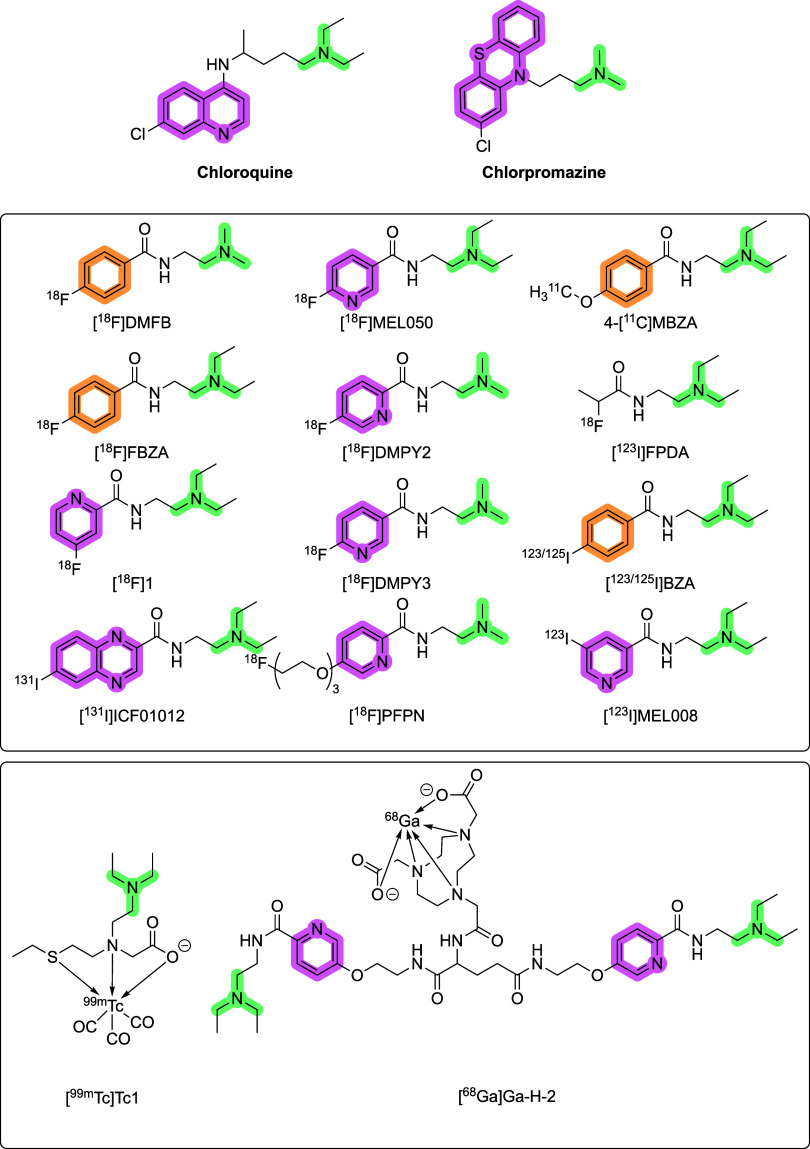
Structures of chloroquine and chlorpromazine (top) and selected melanin-binding SPECT and PET probes (bottom). Common structural motifs highlighted (orange: aromatic; magenta: heteroaromatic; green: tertiary amine).

**Figure 4:**
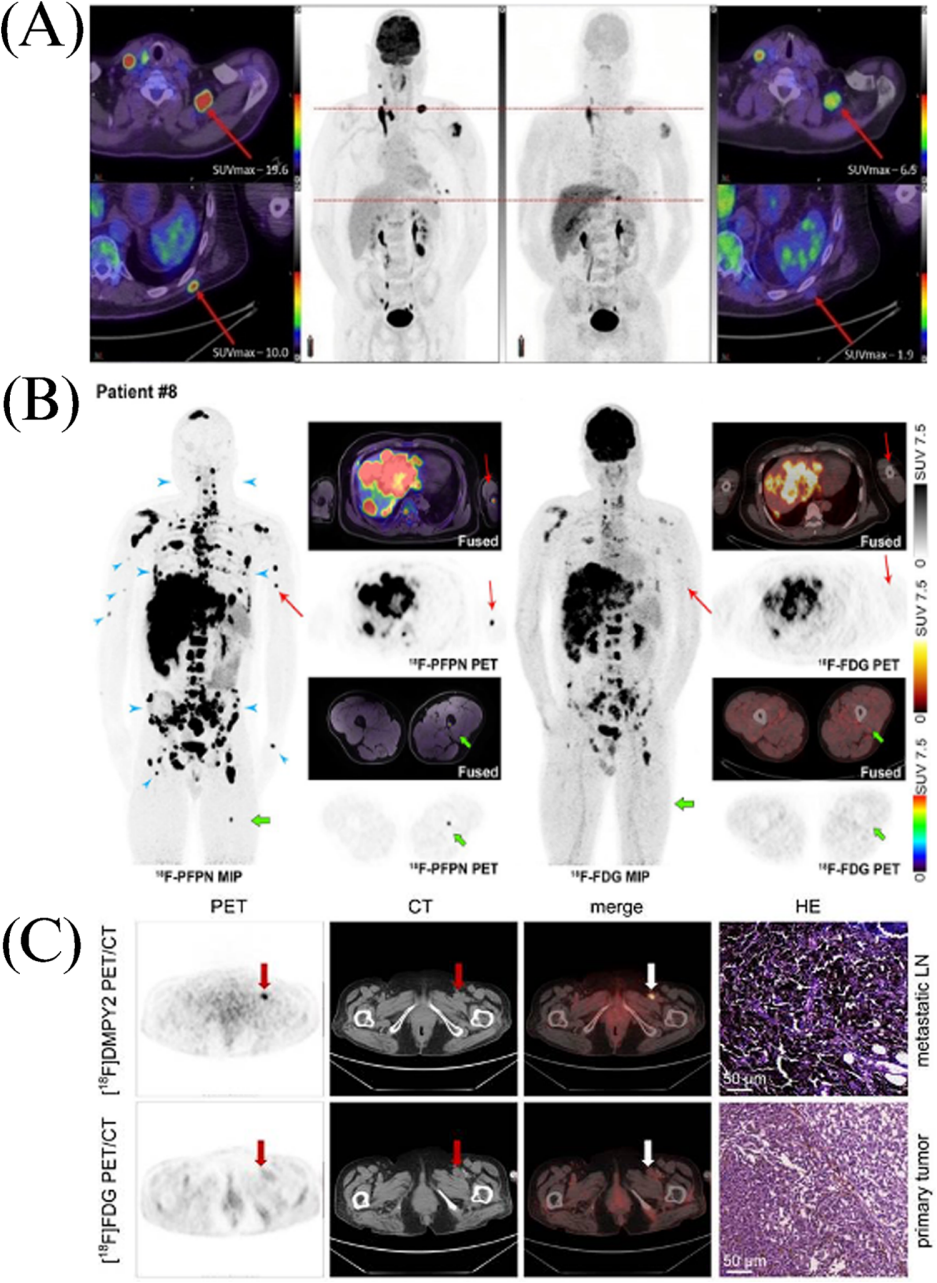
Comparative PET Images of melanin-specific tracers and [^18^F]­FDG in patients with metastatic melanoma. (A) PET images of [^18^F]­MEL050 (left) and [^18^F]­FDG (right); reproduced from ref 111 (Ware et al., 2025), licensed under a CC BY 4.0 license. (https://creativecommons.org/licenses/by/4.0/). (B) PET images of [^18^F]­PFPN and [^18^F]­FDG in a patient with metastatic choroidal melanoma; reproduced from ref 45 (Zhang et al., 2022). Copyright, SNMMI. (C) PET, CT and PET/CT images of a patient with metastatic melanoma, imaged with either [^18^F]­DMPY2 or [^18^F]­FDG; reproduced from ref 30 (Yang et al., 2025), licensed under a CC BY 4.0 license.

### Non-Melanogenesis-Related Targets in Melanoma

Apart from the melanoma-specific antigens, found in the melanogenic pathway, several other tumor-antigens have been identified that are overexpressed in melanoma. These include the melanoma cell adhesion molecule (MCAM, also known as CD146 or MUC18),^115^ the vascular cell adhesion molecule 1 (VCAM1)^116^ as well as various integrins, particularly the well-studied α_V_β_3_ and α_4_β_1_ (also known as very late antigen 4)^117,118^ and the fibroblast activation protein (FAP).^119^ Of these, only inhibitors of FAP (FAPi) have been assessed clinically.^120,121^

### Melanoma Cell Adhesion Molecule

What was originally identified as a cell adhesion molecule specific to melanoma, has since emerged to be a frequently overexpressed protein in many tumors and endothelial cells. As such, the original name (MCAM) is rarely used today and in its stead, it is typically referred to as MUC18 or CD146.^115^ It is not just a cell adhesion molecule but has been found to act as a cell surface receptor with affinity to a variety of growth factors and other proteins.^122^ As such, CD146 has been suggested to be involved in various steps of metastasis (both lymphatic and vascular). CD146 has so far only been investigated in preclinical settings, by using the zirconium-89 labeled radioimmunoconjugates [^89^Zr]­Zr-IP150 and [^89^Zr]­Zr-DFO-Ab253.^115,123^ Results in a number of xenograft models were promising and further development of radiopharmaceuticals against this antigen for use in a number of cancers is warranted.

### Integrins

The two well-studied integrins very-late antigen 4 (VLA4, integrin α_4_β_1_) and integrin α_V_β_3_ have been investigated in detail for a wide range of cancers, including melanoma.^117,118^ VLA4 targeting with derivatives of the peptidomimetic LLP2A have been explored successfully in various models of melanoma and proven to be potential agents for their diagnosis and treatment.^117,124,125^ Pun et al. developed a LLP2A derivative featuring a short PEG4-linker, and a lysine residue as branched linker motif that terminates into a DOTAGA chelator through the ε-amino group and a *para*-iodophenyl butyric acid motif, used as a serum-albumin binder through the α-amino group.^126^ The addition of the serum albumin binding motif resulted in a marked increase in circulatory half-life, uptake and, importantly, therapeutic efficacy.^126^

### Fibroblast Activation Protein

More recently, inhibitors of the fibroblast activation protein (FAPI) have gained interest for the diagnosis and treatment of a range of human malignancies.^127^ These target not the cancer cells themselves, but cancer-associated fibroblasts within the tumor microenvironment (TME).^119–121^ In melanoma, overexpression of this protein is associated with poor clinical outcomes.^128^ Despite this association, several aspects of the underlying mechanism remain unclear, although the effects on remodeling of the extracellular matrix, angiogenesis and immunosuppression are well-documented.^128,129^ Several clinical trials exploring radiopharmaceuticals based on small-molecule FAPIs are currently ongoing and have demonstrated the potential for staging and monitoring of malignant melanoma.^119,120^ Although none of these specifically focus on rare melanoma subtypes, a case report of a 56-year-old man with mucosal melanoma of the nasal cavity has been described in the literature.^121^ Beyond its use as an imaging agent, FAPIs may prove to be useful as therapeutic radiopharmaceuticals, with various agents being in different stages of preclinical and clinical development, including in the context of melanoma.

### Future Directions

A considerable amount of preclinical and clinical research has been conducted toward the development of radiopharmaceuticals for the diagnosis and treatment of melanoma. Beyond the targets discussed in this review, some limited research has been conducted into other targets of relevance in melanoma and other cancers, including GD2, IGFR2 and various amino acid transporters. Table 1 highlights all actively recruiting clinical studies or those of unknown status utilizing targeted radiopharmaceuticals other than FDG in malignant melanoma. While direct radiation damage to target cells has historically been attributed to the therapeutic potential of ionizing radiation, the emerging data on its effects on the tumor microenvironment (TME) has significantly reshaped the field.^48^ Specifically, the activation of innate and adaptive immune responses to radiation treatments have recently been investigated in more detail and paved the way to combination treatments, featuring radiation and immune-checkpoint inhibitors (ICIs).^103^ Especially in the light of the various resistance mechanisms melanoma cells display toward chemo- and immunotherapeutics, similar combination approaches may prove to be promising strategies. Another area, that may require further research is the imaging and treatment of amelanotic lesions characterized by, for instance, mutated variants of MC1R. In addition to MC1R, other members of the GPCR family may be relevant targets. Previous research has demonstrated the expression of SSTRs 1–5 in melanoma and the possibility of imaging SSTR2 expression in this disease context, although expression levels in many patients are low.^130^ In addition, the dopamine receptor D2R was demonstrated to be highly expressed in uveal melanoma.^131^ Despite this, drugs or imaging agents targeting this receptor are currently limited to diagnostic purposes due to endogenous D2R expression in the basal ganglia. Nevertheless, investigations into the interaction and signaling overlaps between the various GPCRs may offer a promising avenue for developing new therapies against melanoma. The recent developments with agents targeting antigens that are not related to the melanogenic pathway, such as FAPIs may be of high relevance in this context. The poor prognoses of patients with rare subtypes, and the difficulties associated with their diagnosis and treatment warrant further development across the entire spectrum of therapeutic options, and recent developments have highlighted that radiopharmaceuticals can be a vital part of these.

**Table 1:** Overview of Clinical Trials Actively Recruiting, Recently Terminated or of Unknown Status at the Time of Writing, the Tested Radiopharmaceutical and the Respective Target Antigen

clinical trial	status	radiopharmaceutical	target
NCT01620749	Unknown (last update 2012)	[^18^F]MEL050	Melanin
NCT03033485	Unknown (last update 2017)	[^18^F]-P3BZA	Melanin
NCT05645484	Unknown (last update 2023)	[^18^F]PFPN	Melanin
NCT02307630	Active, not recruiting	[^124^I]Hu3F8	GD2
NCT01266096	Active, not recruiting	[^124^I]cRGDY-PEG-dots	Integrin α_V_β_3_
NCT05432193	Terminated 2025	[^177^Lu]PNT6555	FAP
NCT03746431	Terminated 2026	[^225^Ac]FPI-1434	IGFR2
NCT05655312	Recruiting	[^212^Pb]VMT01 and Nivolumab	MC1R/PD-1
NCT05496686	Recruiting	[^225^Ac]MTI-201	MC1R
